# Management of Orbital and Periorbital Venous Malformation

**DOI:** 10.3389/fsurg.2017.00027

**Published:** 2017-05-29

**Authors:** Lara A. Benoiton, Kenneth Chan, Frederica Steiner, Trevor FitzJohn, Swee T. Tan

**Affiliations:** ^1^Centre for the Study & Treatment of Vascular Birthmarks, Wellington Regional Plastic, Maxillofacial and Burns Unit, Hutt Hospital, Wellington, New Zealand; ^2^Department of Ophthalmology, Hutt Hospital, Wellington, New Zealand; ^3^Gillies McIndoe Research Institute, Wellington, New Zealand

**Keywords:** venous malformation, orbital, periorbital, management, treatment

## Abstract

**Background:**

To review our management of common venous malformation (VM) affecting the orbit and/or periorbital area.

**Methods:**

Consecutive patients with orbital and/or periorbital VM were identified from our vascular anomalies database. Demographic details of the patients, anatomic site(s) affected, symptoms and signs, presence of a family history of VM, and types of treatment(s) were collected, supplemented by chart review.

**Results:**

A total of 24 patients’ age 1–68 (mean, 30) years with orbital and/or periorbital VM presented with cosmetic concerns (*n* = 17, 71%), distensibility (*n* = 15, 63%), pain (*n* = 9, 38%), diplopia (*n* = 4, 17%), and spontaneous thrombosis (*n* = 1, 8%). The VM caused globe dystopia (*n* = 13, 54%), enophthalmos (*n* = 6, 25%), proptosis (*n* = 3, 12%), exotropia (*n* = 3, 12%), and pseudoptosis with visual obstruction (*n* = 3, 13%). A total of 11 (46%) patients were managed conservatively. 13 (54%) patients underwent active treatment. Ethanol sclerotherapy (ES) was performed in six patients with extensive facial VM associated with orbital/periorbital involvement, resulting in symptomatic improvement in five patients, one of whom developed skin necrosis and another patient developed reduced infraorbital nerve sensation. Surgery was performed for localized lesion (*n* = 3, 23%), for extensive lesions (*n* = 4, 31%) and as an adjunct to ES (*n* = 6, 46%) resulting in symptomatic improvement in all patients. One patient required correction of lower lid ectropion.

**Conclusion:**

Orbital and/or periorbital VMs are heterogeneous, and management needs to be individualized. Surgery is used for localized lesions aiming for complete excision, as a debulking procedure for extensive orbital/periorbital VM when ES was not possible, or following ES for extensive facial VM with orbital and/or periorbital involvement.

## Introduction

Venous malformation (VM) is the most common type of vascular malformation that affects approximately 1% of the population with 40% occurring in the head and neck region (1). It is mostly sporadic with 1.2% being familial (2). A mutation in the TIE-2 gene, a receptor for angiopoietin 1, has been identified in familial (3), and in up to 50% of sporadic (4), VM cases.

Venous malformation is present at birth and increases in size proportionally with the child (5). It may suddenly expand in response to hormonal changes or trauma (6). It may present later in life (6) with pain, swelling, ulceration, loss of function, and deformity (6, 7). Symptoms specific to orbital and/or periorbital VM include reduced visual acuity, diplopia, globe dystopia, and distensibility, where the lesion increases in size on head dependency or Valsalva maneuver (8, 9).

Histologically VM consists of a network of anomalous ectatic venous channels lined with flat endothelium (10, 11). Deficiency of smooth muscle cells and elastic tissue within the walls of the affected veins leads to sequential “blowout” of the channels as the walls become progressively thinner (6). Venous stasis within these channels leads to thrombosis and subsequent thrombophlebitis. Dystrophic calcification within the thrombus results in the formation of phleboliths (6, 12).

Diagnosis of VM is made by careful history and physical examination and confirmed by appropriate imaging, with magnetic resonance imaging (MRI) with gadolinium contrast being the single most useful investigation (13, 14). VM appears isotense with muscle on T1-weighted sequence, and uniform enhancement with gadolinium. It appears uniformly hypertense on T2-weighted sequence. Flow voids represent phleboliths within the dilated channels (6).

Venous malformation has been categorized into common VM, familial cutaneous-mucosal VM presenting with multiple lesions affecting the skin and mucosa (15), blue rubber bleb syndrome (Bean syndrome) affecting the skin, soft tissue, and gastrointestinal tract (16), glomuvenous malformations affecting the skin with nodular or plaque-like lesions, sometimes with a cobblestone appearance (15), cerebral cavernous malformations that may present with seizures, headaches, or focal neurological deficit (17), or be part of a mixed lesion (15) or limb altered growth syndrome (18).

The heterogeneous nature of common VM affecting different types of tissues and involving various topographic regions necessitates individualized treatments (19). There is currently limited data on the management of orbital and/or periorbital VM (1, 6, 8). This study reviewed our management of common VM affecting the orbit and/or periorbital area.

## Materials and Methods

Consecutive patients with common VM were identified from the prospectively maintained vascular anomalies database at the Centre for the Study & Treatment of Vascular Birthmarks between 1996 and 2015. All patients with orbital/periorbital VM were selected for this study, which was carried out with an exemption from Hutt Valley DHB internal review board. Written informed patient or parental consent was obtained for publication of their images.

Demographic details of the patients, anatomic site(s) affected, symptoms and signs, family history of VM, and types of treatment(s) were recorded, supplemented by information from the medical records. The location of the VM was classified as either orbital, where the lesion was confined to the orbit; periorbital, where the lesion affected the periorbital structures; or a combination of orbital and periorbital involvement. Symptomatic improvement and complications associated with treatment were analyzed.

## Results

Of the 1,907 consecutive patients with vascular anomalies, 316 patients had common VM, with orbital and/or periorbital involvement in 24 (0.8%) patients. A total of 15 (62%) patients were females, and 9 (38%) were males, with a mean age of 30 (range, 1–68) years. A total of 12 (50%) patients had combined orbital and periorbital involvement, 6 (25%) patients had periorbital lesions only, and 6 (25%) had a lesion confined to the orbit. Presenting symptoms included cosmetic concerns (*n* = 17, 71%), distensibility (*n* = 15, 63%), pain (*n* = 9, 38%), and diplopia (*n* = 4, 17%). VM caused globe dystopia (*n* = 13, 54%), enophthalmos (*n* = 6, 25%), proptosis (*n* = 3, 13%), exotropia (*n* = 3, 13%), and pseudoptosis with visual obstruction (*n* = 3, 13%) (Table 1).

**Table 1:** **Orbital and/or periorbital venous malformation in 24 patients**.

	*N* (%)
**Gender**	
Female	15 (63)
Male	9 (37)
**Ethnicity**	
European	19 (79)
Maori	2 (8)
Asian	2 (8)
Iraqi	1 (4)
**Location**	
Orbital	6 (25)
Periorbital	6 (25)
Orbital and periorbital	12 (50)
**Symptoms and signs**	
Cosmetic concerns	17 (71)
Distensibility	15 (63)
Globe dystopia	13 (54)
Pain	9 (38)
Enophthalmos	6 (25)
Diplopia	4 (17)
Pseudoptosis with visual obstruction	3 (13)
Proptosis	3 (13)
Spontaneous thrombosis	1 (8)

The patients were managed through our multidisciplinary vascular anomalies clinic. Most patients had undergone imaging on referral. A total of 19 patients had an MRI with gadolinium enhancement, and 1 patient had a contrast enhanced computed tomography. Management was determined by the extent and location of the VM and the patients’ symptoms. Conservative management was advised if there were no or minimal symptoms and/or according to the patient’s preference. A total of 11 (46%) patients were managed conservatively with the mean length of follow-up of 2 (range, 1–26) years. Ethanol sclerotherapy (ES) was performed on six (25%) patients with extensive facial VM with orbital/periorbital involvement (Table 2). The patients required one to seven (mean, 2) ES sessions resulting in improvement of symptom in five (83%) patients. One patient developed reduced infraorbital nerve sensation, and another patient had skin necrosis of the upper lip requiring excision and direct closure. A total of 13 (54%) patients underwent active treatment with complete surgical excision for localized lesions in 4 (31%) patients, as a surgical debulking procedure for extensive orbital/periorbital lesions in 3 (23%) patients and as an adjunct to ES in 6 (46%) patients (Table 2). All 13 patients had symptomatic improvement following surgery. One patient developed ectropion of the lower lid postoperatively requiring surgical correction (*n* = 1, 8%). One (8%) patient suffered amblyopia and impaired visual development due to prolonged occlusion of the visual axis by a large orbital and periorbital VM (Case 3). None of the patients in this cohort, either managed conservatively or having undergone ES and/or surgical intervention, suffered reduction in visual acuity during the follow-up period. None of the patients in this cohort sustained iatrogenic strabismus or globe dystopia following ES and/or surgery.

**Table 2:** **Management of orbital and/or periorbital venous malformation in 24 patients**.

Management	*N* (%)
Conservative management	11 (46)
Active treatment	13 (54)
Complete excision for localized lesion	4 (31)
Surgical debulking for extensive lesion	3 (23)
Surgical debulking following ethanol sclerotherapy for extensive lesion	6 (46)

Representative cases of orbital/periorbital VM who underwent active treatment are presented below.

### Case Reports

#### Case 1

**Figure 1:**
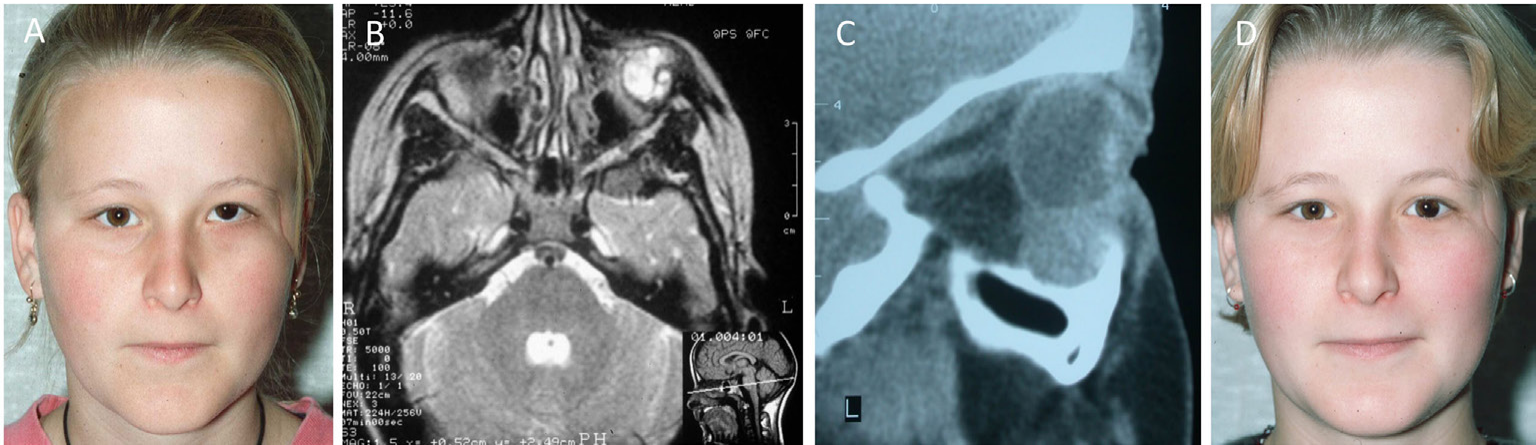
**A 17-year-old female with a left orbital venous malformation causing ocular dystopia**. The visible temporal scar resulted from a previous failed attempt of excision elsewhere. **(A)** A n MRI **(B)** and a CT scan **(C)** showed a 2 cm × 2 cm soft tissue mass on the floor of the left orbit displacing the globe superiorly and a bony ridge at the inferior orbital margin **(C)**. The lesion was excised through a subciliary incision with removal of the bony lip and reduction of the hypertrophic orbital floor, restoring the position of the globe. Photograph 2 years postoperatively **(D)**. Reproduced with permission from the British Journal of Oral and Maxillofacial Surgery (5).

A 17-year-old female was referred with a left intra-orbital VM causing 5 mm vertical ocular dystopia, and a visible temporal scar resulted from a previous failed attempt of excision elsewhere (Figure 1A). The original CT scan showed a 2 cm × 2 cm soft tissue mass on the floor of the left orbit displacing the globe superiorly, and a bony ridge at the inferior orbital margin (Figure 1C). MRI showed that the orbital lesion was intimately related to the inferior rectus muscle and displacing it slightly medially, although there was a clear plane between these structures. The lesion was hyperintense on T2-weighted images (Figure 1B) with marked gadolinium contrast enhancement on T1-weighted sequence. The lesion was excised through a subciliary incision with removal of the bony lip and reduction of the hypertrophic orbital floor, restoring the position of the globe. The histology confirmed a VM. At follow-up 4 years after surgery, there was no clinical or MRI evidence of recurrence of the VM. The left globe remained in a satisfactory position with no visual dysfunction. The patient developed lower lid ectropion postoperatively requiring surgical correction with satisfactory outcome (Figure 1D).

#### Case 2

**Figure 2:**
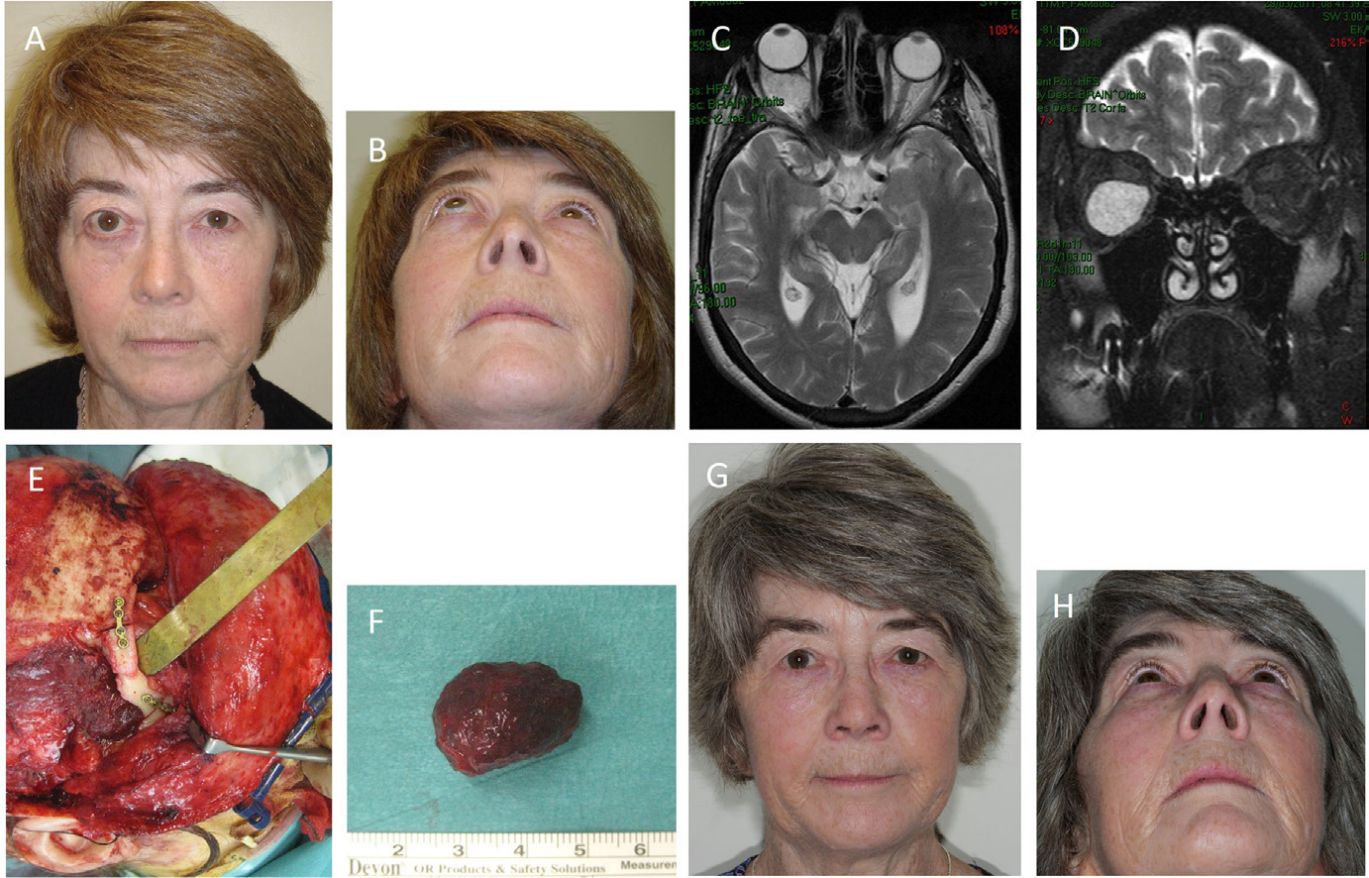
**A 62-year-old female with right eye proptosis resulting from an intraconal venous malformation (VM) (A,B)**. Axial **(C)** and coronal **(D)** T2-weighted magnetic resonance imaging showing a 2.8 cm × 2.4 cm VM within the right orbital cone. The lesion **(E)** was removed through a lateral orbitotomy *via* a coronal incision. The orbitotomy was plated **(E)**. Photograph of the lesion following removal **(F)**. The position of the right globe was restored and remained satisfactory with no visual dysfunction 5 years postoperatively **(G,H)**.

A 62-year-old female presented with right eye proptosis resulting from an intraconal VM (Figures 2A,B). MRI showed a 2.8 cm × 2.4 cm VM within the right orbital cone (Figures 2C,D). The lesion was excised through lateral orbitotomy *via* a coronal incision (Figures 2E,F) restoring the globe to a normal position with no visual dysfunction and remained satisfactory when reviewed 5 years later (Figures 2G,H) when a repeat MRI scan revealed no evidence of recurrence.

#### Case 3

**Figure 3:**
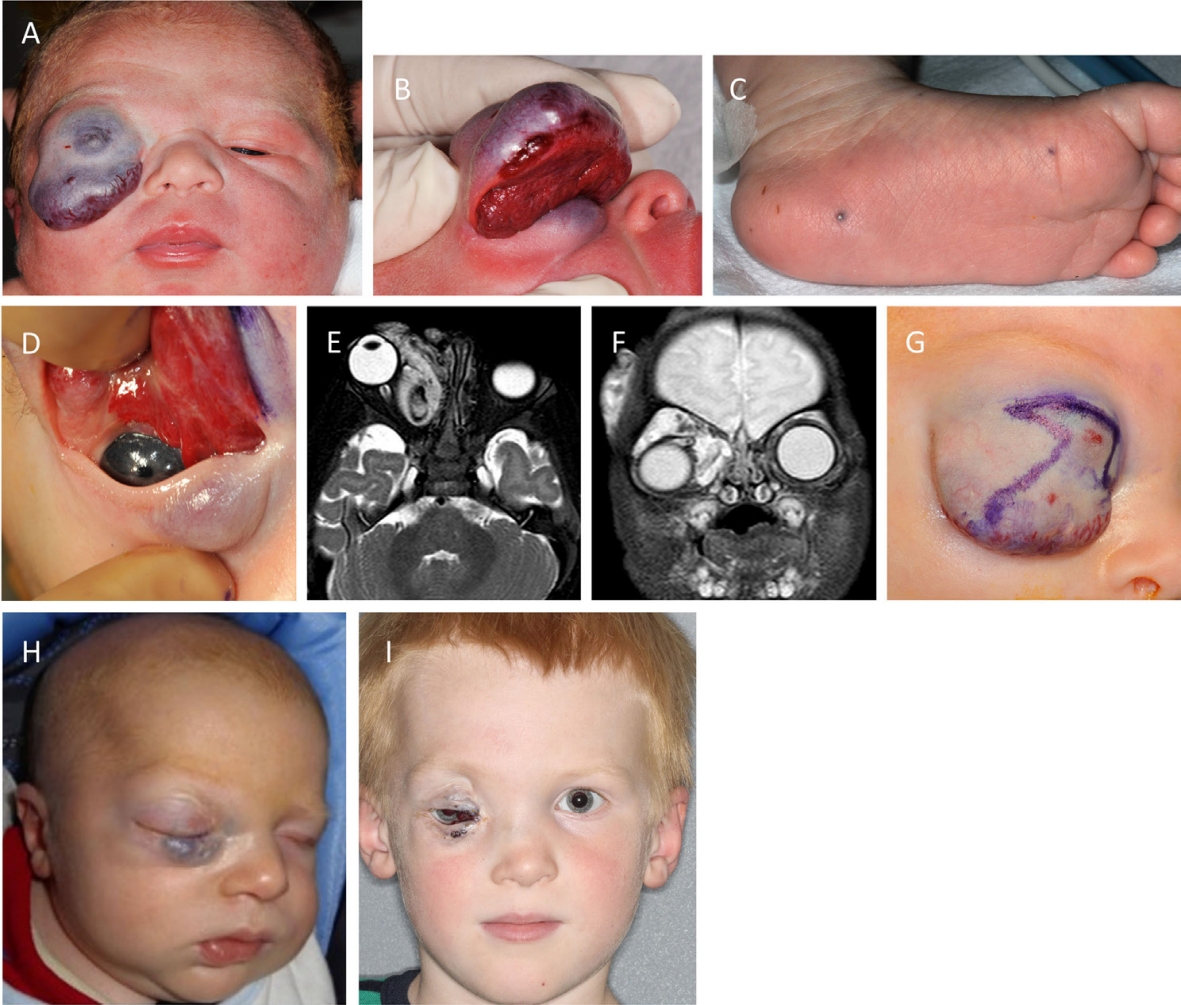
**A newborn male with an extensive orbital and periorbital venous malformation (VM) (A,B) and small cutaneous lesions in the feet (C)**. The lesion massively expanded the upper lid, occluded the visual axis, displaced the globe, and involved the lower lid to a lesser extent **(A,B,D)**. Axial **(E)** and coronal **(F)** T2-weighted magnetic resonance imaging showing extensive orbital/periorbital involvement with intracranial communication, and affecting the temporal area. A debulking procedure was performed **(G)** with significant reduction of the involved upper lid with improvement postoperatively **(H)**. There was gradual expansion of the VM required further debulking procedure 28 months later. The globe remained displaced with minimal vision **(I)**.

A newborn male presented with an extensive orbital and periorbital VM (Figures 3A,B) and small cutaneous lesions in the feet (Figure 3C). The periorbital lesion massively expanded the upper lid, occluded the visual axis, displaced the globe inferiorly and laterally, and involved the nose, glabella and lower lid to a lesser extent (Figures 3A,B,D). Initial ultrasonography and MRI confirmed extensive involvement of the orbit, upper and lower eyelid. The findings suggested the possibility of an infantile hemangioma, although the diagnosis was guarded as gadolinium contrast was withheld because of its associated risk of renal failure in a newborn. A trial of propranolol was commenced with equivocal results. The lesion was complicated by profuse bleeding requiring blood transfusions. A subsequent MRI with gadolinium contrast demonstrated an extensive orbital and periorbital VM with draining to the cavernous sinus through the dilated ophthalmic veins, and a component in the temporal area (Figures 3E,F). Propranolol was discontinued, and a debulking procedure (Figure 3G) was performed with significant reduction of the involved upper lid (Figure 3H). Histology confirmed a VM. A frontalis sling was performed 3 weeks later, and amblyopia therapy was instigated. There was gradual expansion of the VM required further debulking procedure 28 months later. The globe remained displaced with minimal vision (Figure 3I).

#### Case 4

**Figure 4:**
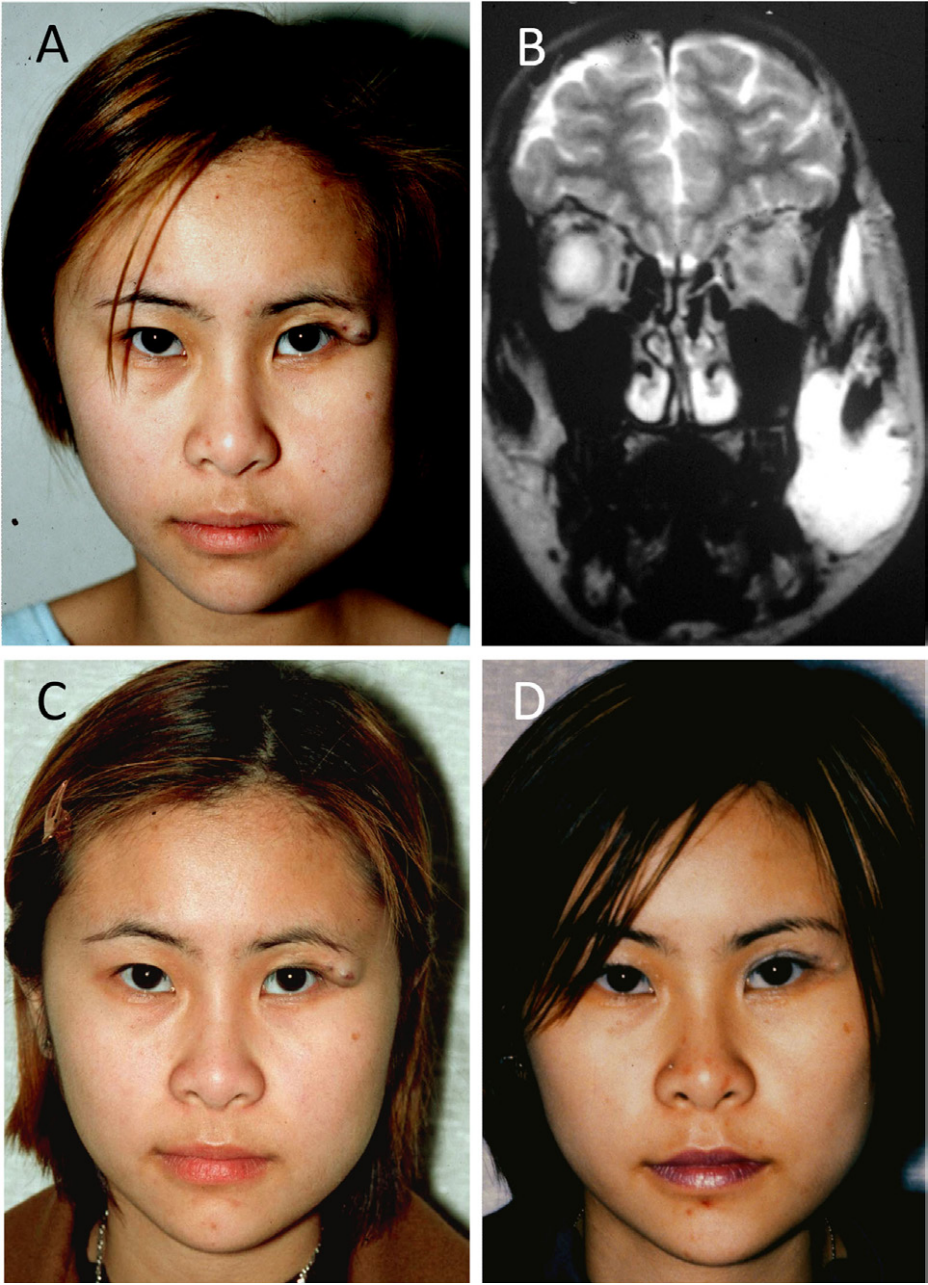
**A 15-year-old female with a venous malformation (VM) affecting her left cheek and peri-orbital area (A)**. A T2-weighted magnetic resonance imaging showing the VM within the left buccal space, extending into the temporal region, deep to the temporalis muscle **(B)**. Ethanol sclerotherapy of the VM led to improved facial symmetry **(C)**. The residual VM over the lateral canthus was debulked and remained unchanged over 2 years **(D)**. Reproduced with permission from the Australian and New Zealand Journal of Surgery (27).

A 15-year-old female with a VM affecting her left cheek and periorbital area (Figure 4A). The periorbital component was treated with surgical debulking elsewhere previously. MRI showed the VM within the left buccal space, extending into the temporal region, deep to the temporalis muscle (Figure 4B). ES of the VM was performed leading to marked improvement of the facial symmetry (Figure 4C). The residual VM over the lateral canthus was debulked and the residual lesion remained unchanged over 2 years (Figure 4D).

## Discussion

Venous malformation affects different topographical regions and tissue types, with varying presentations including swelling/contour deformity, skin discoloration, pain, or loss of function. Categorization of VM can be complex, especially in the head and neck area where it may affect several different tissue types in a single or multiple topographic regions and/or across different subregions. Therefore, treatment cannot be generalized based on a particular topographic region or subregion. The orbit is a typical example where VM can affect orbital tissue (e.g., being intraconal) and/or periorbital tissue such as the conjunctiva and eyelids (19).

Management of orbital and periorbital VM begins with a careful history and physical examination (6, 20). While isolated, localized VM confined to the skin can be diagnosed clinically without imaging, MRI with gadolinium contrast is often required to confirm the diagnosis, define the extent of the lesion, the tissue(s) involved and to assist treatment planning (21, 22). The goals of treatment include reduction of symptoms, improvement of function and/or cosmesis. Management options for orbital/periorbital VM include observation, sclerotherapy, surgical excision, or a combination of these within a multidisciplinary setting (23, 24).

Conservative treatment is recommended for patients with VM causing no or minimal symptoms. ES is preferred for extensive facial VM with periorbital involvement where surgical excision is not an option (25).

Surgery is used as a primary treatment for localized lesions in which complete surgical excision is feasible with low morbidity (6). No postoperative visual complications or recurrence of the VM was observed in this subgroup of patients. While the ultimate aim is complete eradication of the lesion, this may not be possible due to the location and/or the extent of the VM (26). Surgery is used to debulk extensive orbital/periorbital VM when sclerotherapy is not possible because of the location, or as an adjunct to sclerotherapy (6, 22). One patient developed ectropion of the lower lid postoperatively requiring surgical correction. This is consistent with our previous finding of low complication associated with surgery in selected VM patients (6). All our patients achieved symptomatic improvement following surgical treatment.

Visual prognosis for common orbital/periorbital VM is generally good, especially among adults. However, visual prognosis is more guarded in newborn presenting with large orbital/periorbital VM that obstructs the central visual axis, resulting in amblyopia despite early intervention.

This series included a subgroup of patients with extensive facial VM with orbital and/or periorbital involvement. The VM was treated with ES as previously described (27). Ethanol acts as a sclerosant by causing hypertonic dehydration of cells, protein denaturation, thrombosis, and vessel occlusion (25, 27). This leads to an intense inflammatory response producing swelling. Complications of ES include tissue necrosis, nerve damage, pain, swelling, deep vein thrombosis, renal injury, and cardiopulmonary failure (22, 28). One patient developed reduced infraorbital sensation, and another patient developed skin necrosis requiring surgical debridement and direct closure. This was part of the major complications that occurred in 5.6% of patients in a series of 56 patients with VM treated with ES we have reported previously (27).

**Figure 5:**
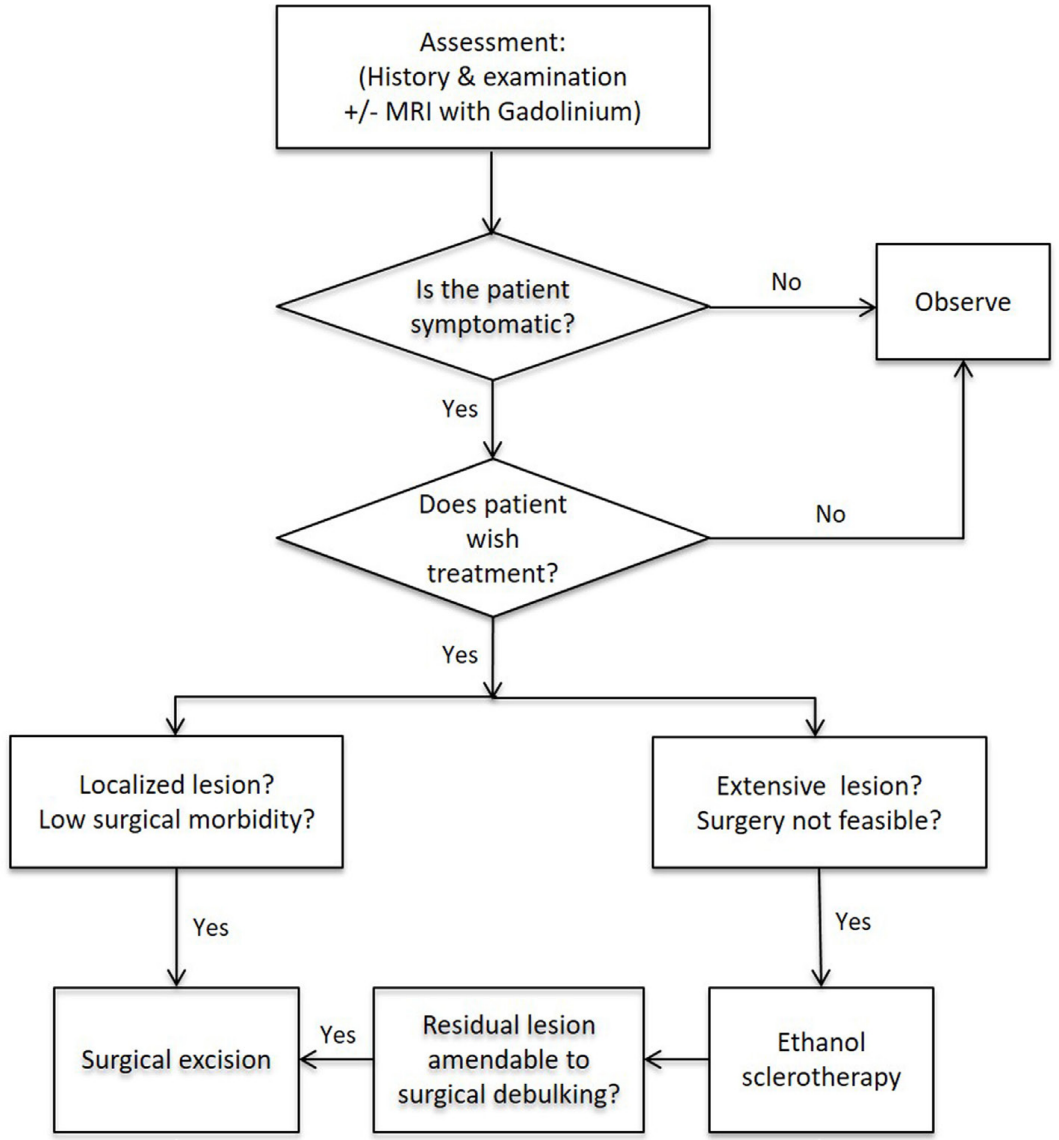
**A proposed management algorithm for orbital and/or periorbital venous malformation**.

Orbital and/or periorbital VMs are heterogeneous and require individualized treatment in a multidisciplinary setting. A flowchart demonstrating the proposed management algorithm is presented on Figure 5.

## Ethics Statement

This study that was carried out with an exemption from Hutt Valley DHB internal review board. Written informed patient or parental consent was obtained for publication of their images.

## Author Note

This paper was presented, in part, at the Oculoplastic Surgeons’ Society Annual Meeting, Bristol, United Kingdom, June 19–21, 2013; and the 85th Annual Scientific Congress of the Royal Australasian College of Surgeons, Brisbane, Australia, May 2–6, 2016.

## Author Contributions

ST formulated the study hypothesis and designed the study. LB, KC, and FS collated the data. LB, KC, FS, and ST analyzed the data. LB and ST drafted the manuscript. All the authors read, commented, and approved the manuscript.

## Conflict of Interest Statement

ST is an inventor of the patent application Treatment of Vascular Anomalies (62/287657), 2016. The authors otherwise declare that the research was conducted in the absence of any commercial or financial relationships that could be construed as a potential conflict of interest.
